# Relationship between COVID-19-specific occupational stressors and mental distress in frontline and non-frontline staff

**DOI:** 10.1016/j.heliyon.2022.e10310

**Published:** 2022-08-18

**Authors:** Megumi Hazumi, Kentaro Matsui, Ayumi Tsuru, Rei Otsuki, Kentaro Nagao, Naoko Ayabe, Tomohiro Utsumi, Michio Fukumizu, Aoi Kawamura, Muneto Izuhara, Takuya Yoshiike, Kenichi Kuriyama

**Affiliations:** a Department of Public Mental Health, National Institute of Mental Health, National Center of Neurology and Psychiatry, Tokyo, Japan; b Department of Sleep-Wake Disorders, National Institute of Mental Health, National Center of Neurology and Psychiatry, Tokyo, Japan; c Department of Laboratory Medicine, National Center Hospital, National Center of Neurology and Psychiatry, Tokyo, Japan; d Department of Psychiatry, Nihon University School of Medicine, Tokyo, Japan; e Department of Regional Studies and Humanities, Faculty of Education and Human Studies, Akita University, Akita, Japan; f Department of Psychiatry, The Jikei University School of Medicine, Tokyo, Japan

**Keywords:** COVID-19, Health care worker, Frontline, Non-frontline, Mental distress

## Abstract

This study investigated the difference in the severity of mental distress and factors contributing to mental distress in frontline and non-frontline healthcare professionals during the coronavirus disease (COVID-19) pandemic. A cross-sectional web-based survey of medical staff collected by snow-ball sampling was performed in Japan in October 2020 using the Kessler Psychological Distress Scale (K6) as an outcome measure for mental distress. Originally developed items asking about the degree of change in psychological and physical burdens, COVID-19-related fear, and experience of discrimination were obtained. The median score of the K6 was 7 in the frontline staff group (n = 86) and 6 in the non-frontline staff group (n = 504), without a statistically significant difference. Multiple regression analyses showed that among the participants, an increase in psychological burden and COVID-19-related fear was significantly associated with mental distress in both groups. Experience of discrimination was significantly associated with mental distress only in the frontline staff group. However, an increase in physical burden was significantly associated with mental distress only in the non-frontline staff group. The results indicate that the factors contributing to mental distress between frontline and non-frontline staff can be different, although the severity of mental distress is comparable between them.

## Highlights

• This study assessed COVID-19 related occupational stressors in medical staff.
• Mental distress associated with fear of COVID-19 in medical staff.
• Discrimination associated with mental distress in frontline staff.
• Physical and psychological burden correlated in non-frontline staff.
• Interventions enhancing interpersonal contact can be helpful for medical staff.

## 1 Introduction

During the coronavirus disease 2019 (COVID-19) pandemic, healthcare workers (HCWs) have been suffering from mental health problems far more than individuals in other occupations (World Health Organization, 2020). According to several meta-analyses, 28%–36% of HCWs have faced mental distress associated with the pandemic (Ching et al., 2021; de Sousa Júnior et al., 2021; Norhayati et al., 2021; Salazar et al., 2020). Mental distress among HCWs can lead to medical errors, poor quality of care, and early retirement, resulting in a risk of patient safety (Anasori et al., 2021; Dall’Ora et al., 2020). Therefore, identifying the factors associated with mental distress among HCWs during the pandemic would be essential for ensuring appropriate healthcare.

Many studies have already reported on the psychological problems in frontline HCWs (Bidzan et al., 2020; Elizabeth et al., 2020; Laukkala et al., 2021; Magill et al., 2020; Sirois and Owens, 2021). Several factors have been found to affect mental distress among frontline staff during the ongoing pandemic. For instance, COVID-19-related fear is considered to aggravate mental distress in frontline staff compared to non-frontline staff. As they are frequently exposed to scenarios that put them at risk of contracting COVID-19, including infection and death (Adams and Walls., 2020), frontline staff are assumed to have a more realistic COVID-19-related fear, which affects their mental distress. Furthermore, the nature of the jobs of frontline staff exposes them to the risk of infecting their families and colleagues (Magill et al., 2020; Muller et al., 2020; Sahashi et al., 2021). These concerns can lead them to avoid contact with their families and colleagues (Fang et al., 2021; Sahashi et al., 2021), which can interfere with the recovery from psychological burden facilitated by warm interpersonal interactions (Muller et al., 2020; Sirois and Owens, 2021). Moreover, experiences of discrimination, which were common in HCWs (Abdel Wahed et al., 2020; Cabarkapa et al., 2020; Rana et al., 2020; Singh and Subedi, 2020), are also suggested to be associated with mental distress among frontline staff, as compared to non-frontline staff. Experiencing discrimination has been reported to be associated with mental health problems such as post-traumatic symptoms, and was more significant in frontline than in non-frontline staff (Zandifar et al., 2020).

Meanwhile, non-frontline staff may also experience mental distress, since they had to provide the same medical care as before the pandemic but with excessive levels of infection control measures (Braquehais et al., 2020; George et al., 2020; Giannouchos et al., 2021; Tiirinki et al., 2020). Non-frontline staff are considered to be overworked, as over half of them having fatigue (Liu et al., 2020; X. Zhang et al., 2020a, Zhang et al., 2020b); additionally, working hours and intensity have contributed to negative psychiatric outcomes in non-frontline staff (Li et al., 2020a, Li et al., 2020b; X. Zhang et al., 2020a, Zhang et al., 2020b), but not in frontline staff (X. Zhang et al., 2020a, Zhang et al., 2020b). Moreover, although several studies have reported that the severity of depression and anxiety is higher in frontline staff than in non-frontline staff (Q. Cai et al., 2020a, Cai et al., 2020b; Z. Z. Cai et al., 2020; De Kock et al., 2021; Noor et al., 2021; X. Zhang et al., 2020a, Zhang et al., 2020b), some studies showed that these symptoms are comparable in both (Sanghera et al., 2020), or rather higher in non-frontline staff (Noor et al., 2021a, Noor et al., 2021b; Norhayati et al., 2021). The differences in results between the studies could be due to varying factors associated with psychological problems in frontline and non-frontline staff. However, the factors related to psychological issues specific to each have not yet been well described.

We hypothesized that mental distress in frontline staff is associated with COVID-19-related fear infection, experience of discrimination, and increased psychological burden, whereas mental distress in non-frontline staff is associated more with a perceived increase in physical burden rather than fear of the disease or experience of discrimination. To verify the above hypothesis, we examined the factors associated with mental distress among frontline and non-frontline medical staff.

## 2 Methods

### 2.1 Design and procedure

This study was conducted within the “Medical workers’ mental health and working conditions in Japan under the COVID-19 pandemic (MEW2-J-COVID) project,” where an anonymous web-based survey on medical institution workers was conducted from October 1 to 30, 2020.

The MEW2-J-COVID project included those who responded to the screening questionnaire in the following manner: (a) those who self-reported as medical workers, and (b) those who self-reported being aged at least 20 years. Among them, those who had worked less than half a month, had not worked in medical institutions, and had been infected by SARS-CoV-2 were excluded from this study.

Participants were collected using snowball sampling. We contacted an unspecified number of medical workers, regardless of their affiliation, and asked them to click on a link to access the questionnaire posted on the website of the National Center of Neurology and Psychiatry (NCNP). Before answering the questionnaire, potential participants were asked to complete a screening questionnaire to confirm that they met the above two inclusion criteria. Those who did not meet these criteria could not respond to the full questionnaire. Google Forms were used to host the questionnaire. Data on participants who discontinued responses and related numbers were not recorded because of the technical specification of the survey form.

### 2.2 Measures

The Kessler Psychological Distress Scale (K6), which measures the severity of mental distress with high validity and reliability (Furukawa et al., 2015; Kessler et al., 2002), was applied as an objective measure. It comprises six items rated on a five-point Likert scale (0) none of the time, (1) a little of the time, (2) some of the time, (3) most of the time, and (4) all of the time. A higher total score indicates severe mental distress. The internal consistency of the K6 in this study was acceptable with a Cronbach’s α coefficient of 0.90.

To classify participants into frontline and non-frontline staff groups, they were asked to answer yes or no to the question: “Are you directly involved in the treatment of patients with COVID-19? “. Those who answered “yes” were classified as frontline and those who answered “no” as non-frontline.

Specific questions to comprehend the perceived change in physical burden (e.g., “How has the ‘physical’ burden of work changed when compared to prior the COVID-19 pandemic?”) and psychological burden (e.g., “How has the ‘psychological’ burden of work changed when compared to prior the COVID-19 pandemic?“) were asked. These questions were evaluated on five levels: (1) markedly decreased, (2) decreased, (3) no change, (4) increased, and (5) markedly increased. To the assess the COVID-19-related fear, we asked participants to respond to “Which of the following describes your fear of COVID-19?” using the three options of (1) no fear, (2) moderate fear, and (3) extreme fear. For experiences of discrimination, we inquired, “After the COVID-19 pandemic, have you experienced discrimination in daycare centers, schools, or from community residents because you are a medical worker?” to which they had to respond with either yes or no. Demographic information such as sex (male or female), age group (20s, 30s, 40s, 50s, 60s, and 70s), and occupation was also noted. Occupation was classified into three variables: physicians, nurses, and others. All questionnaires used in this study were written in Japanese (see Appendix 1 for the English Translation).

### 2.3 Statistical analysis

To confirm the demographic characteristics of the frontline and non-frontline staff, we calculated the median, interquartile range (IQR) and range for continuous variables and the proportion of categorical variables in each group. The differences between the groups were examined using the Mann–Whitney U-test for continuous variables and chi-square test and residual analyses for categorical variables. Multiple regression analyses were performed with K6 as the dependent variable in each group. We included those variables as the independent variables which are potentially related to mental distress, that is, perceived change in physical and psychological burden, COVID-19-related fear, experience of discrimination, and demographic factors such as sex, age group, and occupation (physician, nurse, or others). Multiple regression analyses were performed using the K6 as the dependent variable in each group. We included variables as covariates that are potentially related to mental distress: perceived change in physical and psychological burden, COVID-19-related fear, experience of discrimination, and demographic factors, such as sex, age group, and occupation (physician, nurse, or others) (Adams and Walls., 2020; Magill et al., 2020; Muller et al., 2020; Sahashi et al., 2021; Sirois and Owens, 2021; Abdel Wahed et al., 2020; Cabarkapa et al., 2020; Rana et al., 2020; Singh and Subedi, 2020; Zandifar et al., 2020; Lin et al., 2021; X. Zhang et al., 2020a, Zhang et al., 2020b). The sample size was sufficient to include eight independent variables, given that it was at least ten times the number of independent variables (Wilson Van Voorhis and Morgan, 2007).

Results were considered statistically significant at p < 0.05. All analyses were performed using SPSS ver.25.0.

### 2.4 Ethics

This study was approved by the Ethics Committee of the NCNP in Japan (A2020-044) and performed in accordance with the Declaration of Helsinki. Informed consent was obtained from all participants through a questionnaire. All participants were asked to express their consent to participate by clicking the URL to the questionnaire after reading the explanatory description regarding ethical considerations in this study. They were allowed to discontinue responding to the questionnaire after reading the consent information.

## 3 Results

### 3.1 Characteristics of and differences among the frontline and non-frontline staff

**Figure 1:**
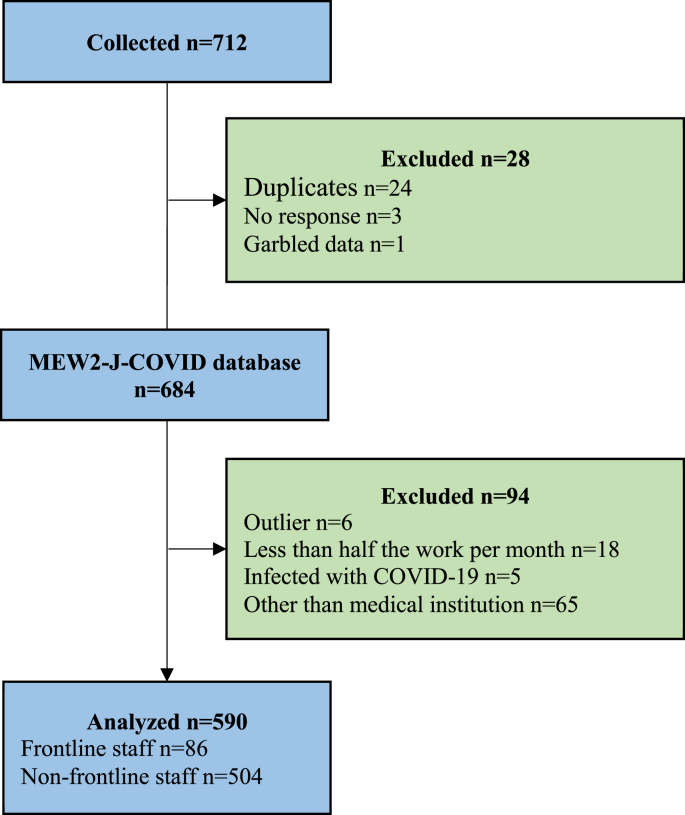
Flow chart.

Of the 684 MEW2-J-COVID participants, 504 were non-frontline staff and 86 were frontline staff (Figure 1). The characteristics and differences between frontline and non-frontline staff are shown in Table 1. In both groups, the largest number of participants was in the 30–39 years age group. There was a significant difference in the percentage of profession between the groups (χ^2^ = 22.37, p < 0.001): the percentage of physicians was significantly larger (adjusted residual = 4.4, p < 0.05) and that of others was significantly smaller (adjusted residual = –3.7, p < 0.05) in frontline staff than in non-frontline staff.

**Table 1:** Demographic characteristics of frontline staff and non-frontline staff (n = 590).

Variables	Non-frontline staff (n = 504)		Frontline staff (n = 86)			
**Sex**						
Male	224	44.4%	46	53.5%	χ^2^ = 2.42	p = 0.12
Female	280	55.6%	40	46.5%		
**Age group (years)**						
20–29	n = 61	12.1%	n = 11	12.8%	χ^2^ = 4.12	p = 0.53
30–39	n = 188	37.3%	n = 37	43.0%		
40–49	n = 169	33.5%	n = 30	34.9%		
50–59	n = 61	12.1%	n = 7	8.1%		
60–69	n = 24	4.8%	n = 1	1.2%		
≥70	n = 1	0.2%	n = 0	0.0%		
**Profession**						
Physician	n = 204	40.5%	n = 57	66.3%	χ^2^ = 22.37	p<0.001
Nurse	n = 149	29.6%	n = 20	23.3%		
Others	n = 151	30.0%	n = 9	10.4%		
**K6 score**	6.0	25	7.0	18	z = -1.79	p = 0.07
**K6 score >12**	n = 15	17.4%	n = 69	13.7%	χ^2^ = 0.85	p = 0.36
**Perceived change in physical burden**						
Increased or remarkably increased	n = 229	45.4%	n = 58	67.4%	χ^2^ = 14.24	p<0.001
Markedly decreased	n = 13	2.6%	n = 5	5.8%	χ^2^ = 25.93	p<0.001
Decreased	n = 45	8.9%	n = 8	9.3%		
Unchanged	n = 216	43.1%	n = 15	17.4%		
Increased	n = 154	30.6%	n = 32	37.2%		
Markedly increased	n = 75	14.9%	n = 26	30.2%		
**Perceived change in psychological burden**						
Increased or remarkably increased	n = 389	77.2%	n = 71	82.6%	χ^2^ = 1.24	p = 0.27
Markedly decreased	n = 2	0.4%	n = 0	0.0%	χ^2^ = 11.26	p = 0.02
Decreased	n = 10	2.0%	n = 2	2.3%		
Unchanged	n = 103	20.4%	n = 13	15.1%		
Increased	n = 239	47.4%	n = 30	34.9%		
Markedly increased	n = 150	29.8%	n = 41	47.7%		
**COVID-19-related fear**						
Not at all	n = 73	14.5%	n = 15	17.4%	χ^2^ = 0.67	p = 0.72
Moderate	n = 343	68.1%	n = 58	67.4%		
Extreme	n = 88	17.5%	n = 13	15.1%		
**Experience of discrimination**						
No	n = 470	93.3%	n = 71	82.6%	χ^2^ = 11.04	p = 0.001
Yes	n = 34	6.7%	n = 15	17.4%		

The median of the K6 score in the frontline and non-frontline staff groups was 7 (IQR = 4–11; range = 2–25), and 6 (IQR = 3–9; range = 1–26), respectively, indicating no significant difference (z = 1.79, p = 0.07). The degree of perceived change in physical and psychological burden was significantly different between the frontline and non-frontline staff groups (χ^2^ = 35.93, p < 0.001; χ^2^ = 11.26, p = 0.002). The residual analysis for physical burden revealed that the percentage of participants who answered “markedly increased” (adjusted residual = 3.5, p < 0.05) was significantly higher and that of participants who answered “unchanged” (adjusted residual = –4.5, p < 0.05) was significantly smaller in frontline staff than in non-frontline staff. The residual analysis for psychological burden revealed that the percentage of participants who answered “markedly increased” was significantly larger (adjusted residual = 3.3, p < 0.05) and that of participants who answered “increased” was significantly smaller (adjusted residual = –2.1, p < 0.05) in frontline staff. The proportion of subjects who experienced discrimination was larger in the frontline staff group (χ^2^ = 11.04, p = 0.001).

### 3.2 Relationship between mental health severity and the difficulties induced by COVID-19

In the multiple linear regression, changes in psychological burden (β = 0.26, p = 0.02), COVID-19-related fear (β = 0.25, p = 0.02), and experience of discrimination (β = 0.21, p = 0.03) were independently significantly associated with K6 scores (Table 2).

**Table 2:** Factors contributing to mental health in frontline staff (n = 86).

Independent variable	B	SE	95% CI	β	p	
Perceived change in physical burden	0.03	0.46	(-0.89–0.95)	0.01	0.95	
Perceived change in psychological burden	1.36	0.65	(0.05–2.66)	0.25	0.04	∗
COVID-19-related fear	1.89	0.81	(0.27–3.51)	0.25	0.02	∗
Experience of discrimination	2.49	1.18	(0.14–4.84)	0.22	0.04	∗
Sex (male = 1, female = 2)	0.98	0.95	(-0.90–2.87)	0.11	0.30	
Age group	-0.51	0.51	(-1.53–0.50)	-0.10	0.32	
Occupation (others as reference)						
Physician	0.70	1.50	(-2.28–3.68)	0.08	0.64	
Nurse	0.88	1.67	(-2.45–4.21)	0.09	0.60	
R^2^ = 0.25, p = 0.003						
Adjusted R^2^ = 0.17						

Note: COVID-19, Coronavirus disease; ∗,p < 0.05.

In the multiple linear regression, changes in physical and psychological burden (β = 0.15, p = 0.004; β = 0.21, p < 0.001) and COVID-19-related fear (β = 0.14, p = 0.001) were independently significantly associated with K6 scores (Table 3).

**Table 3:** Factors contributing to mental health in non-frontline staff (n = 504).

Independent variable	B	SE	95% CI	β	p	
Perceived change in physical burden	0.74	0.24	(0.28–1.20)	0.15	<0.001	∗∗∗
Perceived change in psychological burden	1.32	0.29	(0.75–1.88)	0.22	<0.001	∗∗∗
COVID-19-related fear	1.20	0.35	(0.52–1.87)	0.15	<0.001	∗∗∗
Experience of discrimination	1.22	0.75	(-0.25–2.70)	0.07	0.10	
Sex (male = 1, female = 2)	0.69	0.40	(-0.10–1.47)	0.07	0.09	
Age group	-0.29	0.19	(-0.65–0.07)	-0.06	0.12	
Occupation (others as reference)						
Physician	-1.39	0.46	(-2.29–0.49)	-0.15	<0.001	∗∗∗
Nurse	-1.62	0.49	(-2.58–0.66)	-0.16	<0.001	∗∗∗
R^2^ = 0.21, p < 0.001						
Adjusted R^2^ = 0.20						

Note: COVID-19, Coronavirus disease; ∗∗∗, p < 0.001.

## 4 Discussion

In this cross-sectional survey of HCWs, we examined the hypothesis that mental distress in frontline staff is associated with COVID-19-related fear infection, experience of discrimination, and increased psychological burden, whereas mental distress in non-frontline staff is more associated with a perceived increase in physical burden than COVD-related fear or experience of discrimination. Consistent with our hypothesis, experiences of discrimination were associated with mental distress only in frontline staff. Changes in perceived physical burden were associated with mental distress in non-frontline staff rather than frontline staff. Perceived increase in psychological burden and COVID-19-related fear were associated with mental distress in both frontline and non-frontline staff. The level of mental distress tended to be slightly higher in frontline staff; however, the difference was not significant between these groups. To our knowledge, this is the first study to compare the factors related to the severity of mental distress between frontline and non-frontline staff, respectively, during the COVID-19 pandemic.

The differences in the severity of mental distress between frontline and non-frontline staff remain controversial (Fang et al., 2021; Gemine et al., 2021; Liang et al., 2020; Yi et al., 2021). This may be due to differences in the outcomes used and research settings across studies. In Japan, the risk of facing a shortage of hospital beds due to the spread of COVID-19 and death of COVID-19 patients was not as serious as the risk in other countries at the time of the survey (Ministry of Health Labour and Welfare, 2021; Johns Hopkins University, 2021). The shortage of personal protective equipment (PPE), a factor affecting mental health in HCWs (Gemine et al., 2021; Sirois and Owens, 2021), was not as serious as that in other countries either (Unoki et al., 2020).

In the present study, the proportion of participants who experienced discrimination was higher among frontline staff than among non-frontline staff. Moreover, we found a significant correlation between the experience of discrimination and mental distress only among frontline staff. Although previous studies have indicated the relationship between discrimination against HCWs and their mental health problems during the pandemic (Abdel Wahed et al., 2020; Kandeger et al., 2018; Rana et al., 2020; Singh and Subedi, 2020), no study has compared the differences in the negative effects of discrimination experiences between frontline and non-frontline staff. Among the general population, experiences of discrimination strongly affect mental health among those who recognize the perceived discrimination as appropriate (H. Li et al., 2020a, Li et al., 2020b; Ugidos et al., 2020). Vulnerability to experiences of discrimination may involve stigmatization of oneself, especially among frontline staff, as found in a study that found higher stigma intensity among frontline than non-frontline staff (Zandifar et al., 2020).

Along with our hypothesis, the perceived increase in physical burden was associated with mental distress in non-frontline staff; however, this association was not found in frontline staff. This result is consistent with that of a study that found a relationship between working hours and depression in non-frontline staff (Zhang et al., 2020a, Zhang et al., 2020b). Physical burden, including excessive working hours, intensity, and insufficient human resources, has contributed to mental distress during the COVID-19 pandemic (Braquehais et al., 2020; Zhang et al., 2020a, Zhang et al., 2020b). Excessively higher workloads in non-frontline staff could be a factor contributing to mental health problems in HCWs during COVID-19 (Braquehais et al., 2020; Mo et al., 2020; Song et al., 2020); however, the details of the burden on non-frontline staff were not clearly identified in this study. Another possible reason is that a specific physical burden associated with rapid changes in the medical system not limited to dealing with COVID-19 may have been more serious in the non-frontline than in frontline staff. Both appropriate working hours and working environment should be ensured to reduce the physical workload of non-frontline staff, which could also contribute to reducing medical error and improving quality of care.

No noticeable correlation was found between a perceived increase in physical burden and mental distress among frontline staff. However, the residual analysis compared the changes in physical burden between frontline and non-frontline staff, which indicated that the degree of the perceived increase in physical burden was more prominent in frontline staff. A reason for these results could be the use of PPE. Wearing PPE has been reported to be a physical burden (Unoki et al., 2020). However, it also has a protective effect on psychological burden through the reduction of infection risk (Cabarkapa et al., 2020; Gemine et al., 2021; Sirois and Owens, 2021). Moreover, frontline staff tend to rely on the effectiveness of PPE more strongly than non-frontline staff (Unoki et al., 2020). Another factor is that some frontline staff may have experienced a reduced physical burden despite severe mental distress; sudden, uncontrollable pandemic waves could have strongly affected frontline staff regardless of the perceived change in physical burden (Sirois and Owens, 2021). As only the perceived change in physical burden was investigated in this study, future studies on frontline staff in the pandemic should focus on the intensity of the physical load itself.

In both frontline and non-frontline groups, COVID-19-related fear was related to mental distress. The perceived increase in psychological burden was also associated with mental distress in both groups. Although frontline staff have been previously reported to experience a stronger COVID-19-related fear than non-frontline staff (Lu et al., 2020), both groups reported a similar intensity of COVID-19-related fear in this study. This difference may have been caused by variations in countries and survey periods. While the study by Lu et al. (2020) was conducted in the early stages of the pandemic, the current survey was conducted at a time when the number of infected people in Japan declined (Johns Hopkins University, 2021). It is possible that the participants in this study had more adequate safety measures than those in the prior study, and thus the fear experienced by frontline staff was not as noticeable. Furthermore, cluster outbreaks are very common in hospitals and clinics in Japan (Furuse et al., 2020), which could be a common fear among frontline and non-frontline staff. During the pandemic, personal resilience has protected against fear (Seçer et al., 2020). Given that HCWs tend to avoid interpersonal interaction, a component of resilience (Friborg et al., 2003), due to concerns about infecting their close ones (Simione and Gnagnarella, 2020), maintaining interpersonal interaction can be a feasible and effective intervention target in social support.

Being non-frontline staff, other than a physician or nurse was associated with mental distress, which is consistent with a previous study (Yao et al., 2021). During the pandemic, lack of medical knowledge and short training periods have been shown to be associated with psychological burden (Serrano-Ripoll et al., 2020; Sirois and Owens, 2021). Staff other than doctors and nurses may have less knowledge and it may have been challenging to handle the situation appropriately (M. Zhang et al., 2020a, Zhang et al., 2020b), which could have led to these findings.

This study has some limitations. First, the MEW2-J-COVID data were collected using the snowball technique, which induces sampling bias. However, it can be minimized as we used the “sample seed diversity” method, in which several people collect data from different communities (Kirchherr and Charles, 2018). Second, measurements regarding COVID-19 related burden, fear, and experience of discrimination were not validated because there were few validated scales at the time of data collection. Third, the sample sizes of the frontline and non-frontline groups were different, resulting in differences in the statistical power. The small number of frontline staff in this study could also be a limitation in the interpretation of the results of the multiple regression analysis.

## 5 Conclusion

We found partial but significant differences in the factors associated with mental distress between frontline and non-frontline staff. Although the burden on frontline staff has been emphasized in previous reports, the intensity of mental distress between frontline and non-frontline staff was similar, suggesting that both frontline and non-frontline staff must receive support. Particularly for non-frontline workers, unnoticed increases in workload should be identified, while for frontline workers, measures to mitigate the negative effects of discrimination need to be developed to abate mental distress. Further research is warranted to determine the individual vulnerabilities and promote sustainable protective measures among HCWs during pandemics.

## Declarations

### Author contribution statement

Megumi Hazumi; Kentaro Matsui; Kenichi Kuriyama: Conceived and designed the experiments; Performed the experiments; Analyzed and interpreted the data; Contributed reagents, materials, analysis tools or data; Wrote the paper.

Ayumi Tsuru; Kentaro Nagao; Tomohiro Utsumi; Aoi Kawamura; Takuya Yoshiike: Conceived and designed the experiments; Performed the experiments; Analyzed and interpreted the data; Contributed reagents, materials, analysis tools or data.

Rei Otsuki; Naoko Ayabe; Michio Fukumizu; Muneto Izuhara: Performed the experiments; Analyzed and interpreted the data; Contributed reagents, materials, analysis tools or data.

### Funding statement

This research did not receive any specific grant from funding agencies in the public, commercial, or not-for-profit sectors.

### Data availability statement

Data will be made available on request.

### Declaration of interest’s statement

The authors declare no conflict of interest.

### Additional information

No additional information is available for this paper.
